# Supplementary material for: Paper-Based Electrochemical Biosensors for Voltammetric Detection of miRNA Biomarkers Using Reduced Graphene Oxide or MoS2 Nanosheets Decorated with Gold Nanoparticle Electrodes
Source: Biosensors (Basel). 2021 Jul 13;11(7):236. doi: 10.3390/bios11070236 (PMC8301884; doi:10.3390/bios11070236)
Supplement: Supplementary file 1 [file biosensors-11-00236-s001.zip › biosensors-1260161-supplementary.pdf]

Supplementary Material

Paper-based electrochemical biosensors for voltammetric detection of miRNA biomarkers using reduced graphene oxide or MoS_2 nanosheets decorated with gold nanoparticle electrodes

Hilal Torul [1,a], Ece Yarali [2,a], Ece Eksin [2,a], Abhijit Ganguly [3], John Benson [4], Ugur Tamer [1,*], Pagona Papakonstantinou [3,*] and Arzum Erdem [2,*]

[1] *Department of Analytical Chemistry, Faculty of Pharmacy, Gazi University, 06330 Ankara, Turkey*

[2] *Department of Analytical Chemistry, Faculty of Pharmacy, Ege University, 35100 Bornova, Turkey*

[3] *School of Engineering, Engineering Research Institute, Ulster University, Newtownabbey BT37 0QB, United Kingdom*

[4] *2-DTech, Core Technology Facility, 46 Grafton Street, Manchester M13 9NT, United Kingdom*

Corresponding Authors:

E-mail addresses: arzum.erdem@ege.edu.tr (A. Erdem), p.papakonstantinou@ulster.ac.uk (P. Papakonstantinou), utamer@gazi.edu.tr (U. Tamer)

miRNAs and the base sequences of all oligonucleotides:

miRNA-155, miRNA-21 specific DNA probe, their synthetic target miRNA-155 or miRNA-21 and the other oligonucleotides; non-complementary RNA target, the oligonucleotides having a single base were obtained from (as lyophilized powder) TIB Molbiol (Germany). miRNAs and the base sequences of all oligonucleotides were listed below:

thiol link miRNA-155 specific DNA probe (Probe-1)

5'- SH-ACC CCT ATC ACG ATT AGC ATT AA-3'

miRNA-155 RNA target

5'- UUA AUG CUA AUC GUG AUA GGG GU-3'

miRNA-155 non-complementary sequence (NC)

5'-UGG CAG UGU CUU AGC UGG UUG U-3'

miRNA-155 mismatch sequence (MM)

5'-UUA AUG CUA AUC GUC AUA GGG GU-3'

thiol link miRNA-21 specific DNA probe (Probe-2)

5'- SH-TCA ACA TCA GTC TGA TAA GCT A-3'

miRNA-21 RNA target

5'- UAG CUU AUC AGA CUG AUG UUG A-3'

miRNA-21 non-complementary sequence (NC)

5'- AUG CAU GCA UGC AUG CAU GCA A-3'

miRNA-21 mismatch sequence (MM)

5'-UAG CUU AUC AGA CUC AUG UUG A-3'

The stock solutions of DNA probe and miRNA target were prepared in Tris-EDTA buffer (pH 8.00) and stored in freezer. The stock solutions were diluted using PBS (pH 7.40) solution.

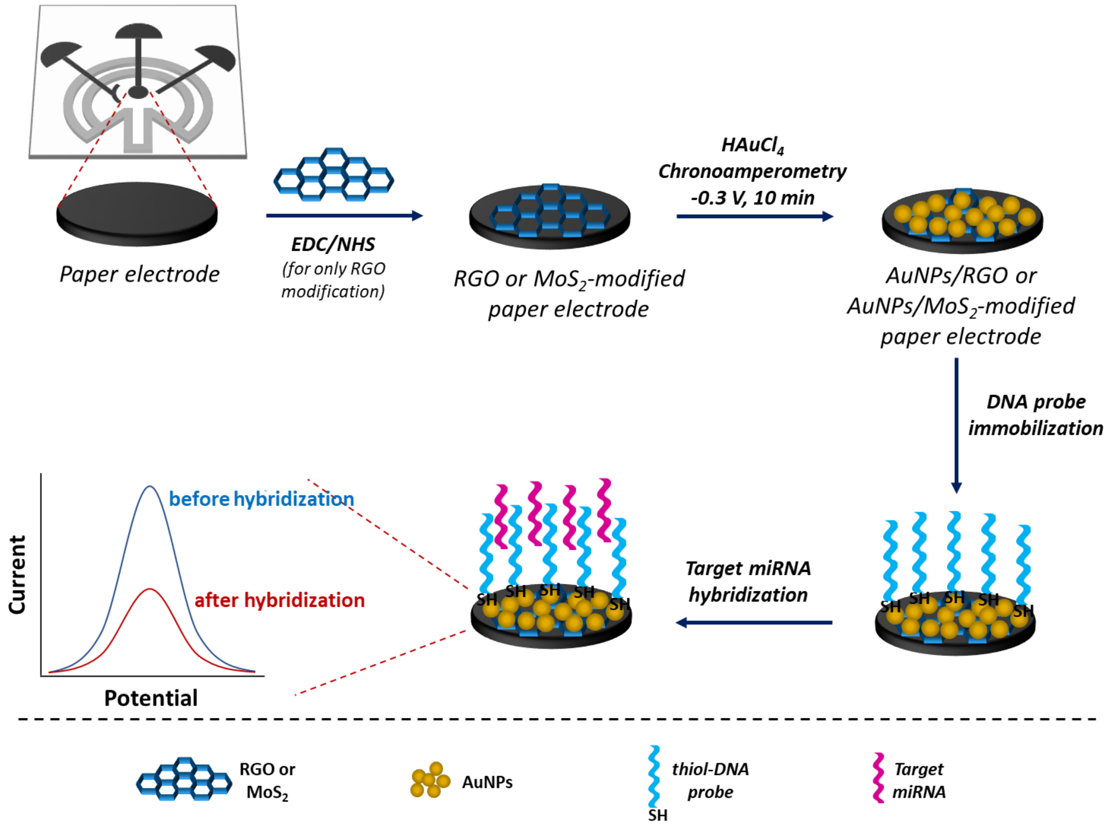

Scheme SI. The schematic illustration of RGO/MoS_2-modified paper electrode assembly fabrication and Probe/miRNA assembling

Results obtained by AuNPs/RGO-modified paper electrode

The electrochemical characterization of RGO-modified paper electrode

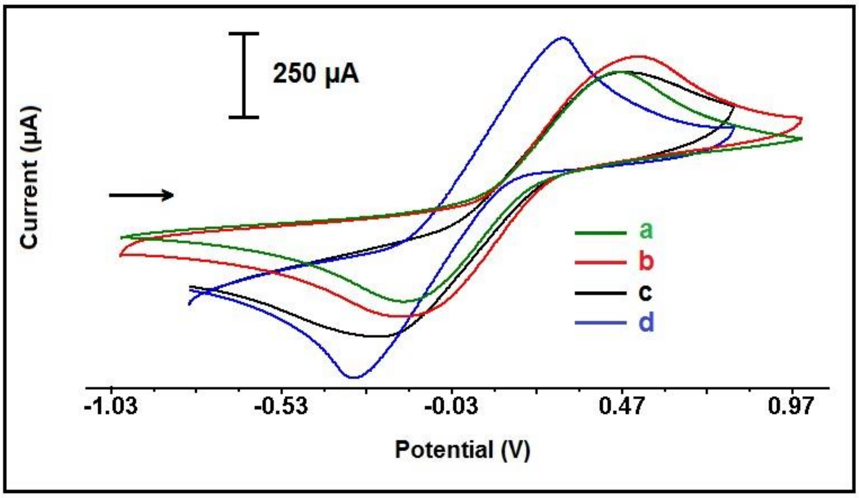

Figure S1. CVs recorded in optimum conditions by using (a) unmodified paper electrode, (b) RGO-modified paper electrode, (c) after activation of RGO-modified paper electrode using covalent agents, (d) after electrodeposition of AuNPs onto the surface of chemically activated and RGO-modified paper electrode in the presence of 50.0 mM potassium ferricyanide in 100.0 mM KCl.

Table S1. The anodic current Ia (μA) and the cathodic current Ic (μA), the relative charge, Qa and Qc of $[Fe(CN)_6]^{3-/4-}$ measured by unmodified, RGO modified, after activation of RGO-modified paper electrode using covalent agents and AuNPs/RGO-modified paper electrode.

	**Ia (μA)**	**Ic (μA)**	**Qa (mC)**	**Qc (mC)**	**A (cm^2)**
**Unmodified paper electrode**	240	281	2.17	3.80	0.020
**RGO-modified paper electrode**	305.9	347.9	3.3	4.41	0.026
**after activation of RGO-modified paper electrode using covalent agents**	211.3	296.5	2.23	4.97	0.018
**AuNPs/RGO-modified paper electrode**	425.6	406	2.53	4.87	0.036

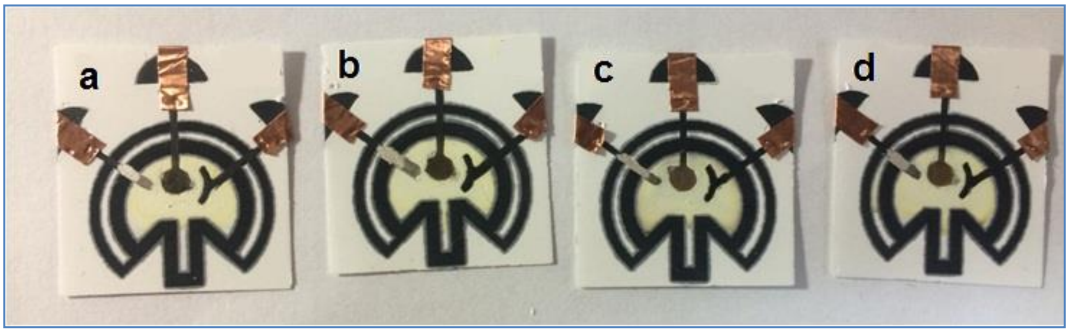

Figure S2. The images of (a) 2.5 mM, (b) 5.0 mM, (c) 10.0 mM, (d) 15.0 mM $HAuCl_4$ deposited RGO-modified paper electrode

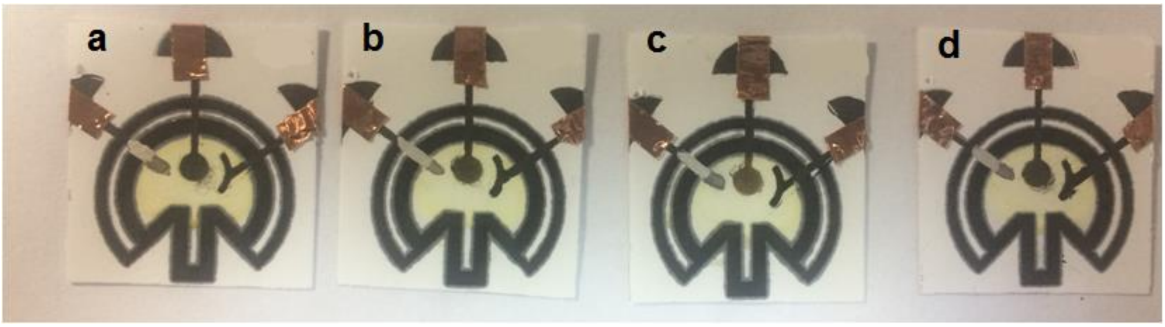

Figure S3. The images of AuNPs/RGO-modified paper electrode after deposition of 5.0 mM $HAuCl_4$ during (a) 5 min, (b) 7 min, (c) 10 min, (d) 15 min.

The effect of probe concentration at hybridization process

The effect of DNA probe concentration upon the hybridization was investigated (Fig. S4). The nucleic acid hybridization was performed between 2.0 µg/mL miRNA-155 target and its DNA probe (Probe-1) at different concentration level from 0.5, 1.0 and 2.0 µg/mL. After immobilization of 0.5 µg/mL Probe-1, the average oxidation signal of $[Fe(CN)_6]^{3-/4-}$ was measured as 32.43 ± 1.12 µA (RSD%, 3.47%, n=3). According to the signal measured in the absence of Probe-1, the highest decrease was obtained in the presence of 0.5 µg/mL Probe-1 as 25.9% and measured as 24.01 ± 6.97 µA (RSD%, 29.01%, n=3). Therefore, 0.5 µg/mL Probe-1 concentration was determined as optimum for further studies.

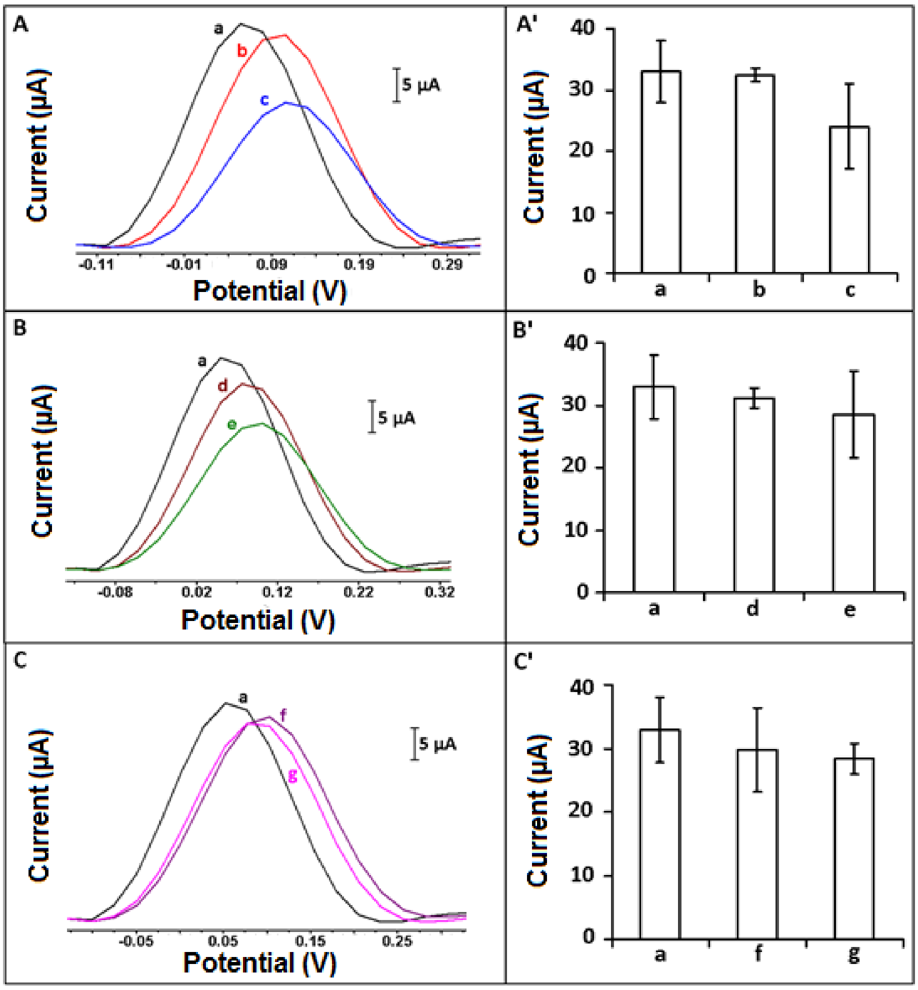

Figure S4. (A) DPVs **(A')** histograms representing (a) AuNPs/RGO-modified paper electrode, (b) 0.5 µg/mL Probe-1 immobilized AuNPs/RGO-modified paper electrode, after hybridization of 0.5 µg/mL miRNA 155 Probe-1 with (c) 2.0 µg/mL miRNA-155 target (n=2). **(B)** DPVs **(B')** histograms representing (d) 1.0 µg/mL miRNA 155 Probe-1 immobilized AuNPs/RGO-modified paper electrode, after hybridization of 1.0 µg/mL Probe-1 with (e) 2.0 µg/mL miRNA-155 target (n=2). **(C)** DPVs **(C')** histograms representing (f) 2.0 µg/mL Probe-1 immobilized AuNPs/RGO-modified paper electrode, after hybridization of 2.0 µg/mL Probe-1 with (g) 2.0 µg/mL miRNA-155 target (n=2).

The effect of probe immobilization time onto the electrode surface upon the hybridization process

For the optimization of probe immobilization time, a short period (i.e. 10 min) which was used in our previous work [35] in contrast to a longer period (i.e. 30 min) were tested. Since the electrode surface dried over 30 min, there is no need to examine a much longer immobilization time in our study.

In the absence of Probe-1, the oxidation signal of $[Fe(CN)_6]^{3-/4-}$ was measured as 32.96 ± 5.12 µA by AuNPs/RGO-modified paper electrode. The average oxidation signal of $[Fe(CN)_6]^{3-/4-}$ was measured as 32.43 ± 1.12 µA (RSD%, 3.47%, n=3) and 25.60 ± 0.14 µA (RSD%, 0.55%, n=3) after immobilization of 0.5 µg/mL Probe-1 during 10 and 30 min, respectively (Fig. S5). According to the signal measured in the absence of Probe-1, the highest decrease at the oxidation signal was obtained in the case of 30

min Probe-1 immobilization as 22 % (Table S2). Thus, 30 min was chosen as optimum probe immobilization time for our further studies.

Table S2. The oxidation signal of $[Fe(CN)_6]^{3-/4-}$ measured before/after 0.5 μg/mL DNA probe immobilization onto the surface of AuNPs/RGO-modified paper electrode during 10 and 30 min and HE% values.

		**I (μA)**	
**AuNPs/RGO-modified paper electrode**		32.96 ± 5.12	**HE%**
**Probe-1 immobilization time (min)**	**10**	32.43 ± 1.12	1.6%
	**30**	25.60 ± 0.14	22%

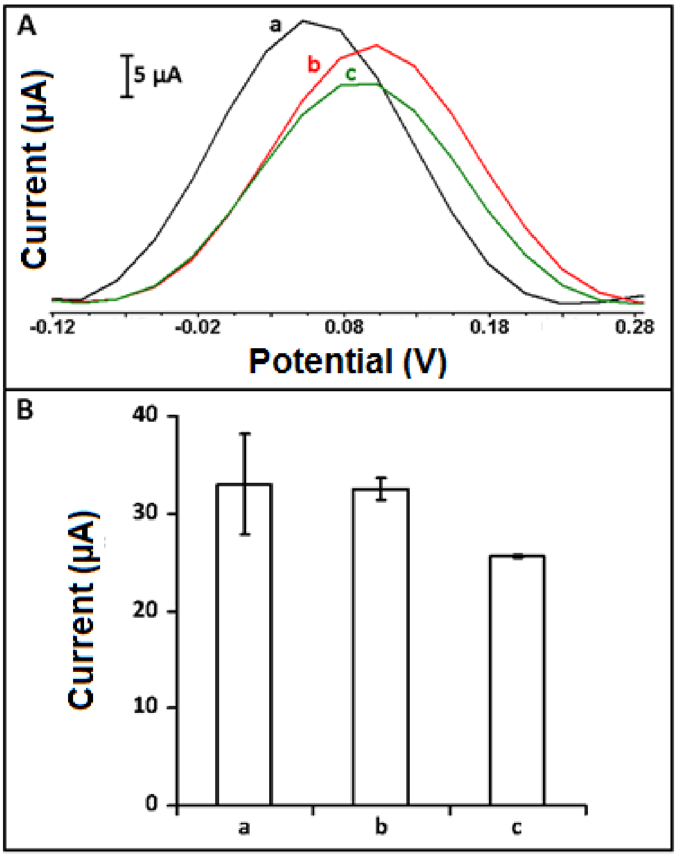

Figure S5. (A) DPVs **(B)** histograms representing 1.0 mM $[Fe(CN)_6]^{3-/4-}$ oxidation signal obtained by (a) AuNPs/RGO-modified paper electrode, after immobilization of 0.5 μg/mL Probe-1 during (b) 10 min (c) 30 min onto the surface of AuNPs/RGO-modified paper electrode (n=3).

The effect of hybridization time upon the hybridization process

For the optimization of hybridization time, a short (i.e. 5 min) which was used in our previous work [35], in contrast to a longer period (i.e. 15 min) were tested.

The hybridization of 0.5 μg/mL Probe-1 and 1.0 μg/mL miRNA-155 target was done during 5 and 15 min (Fig. S6). The average oxidation signal of $[Fe(CN)_6]^{3-/4-}$ was measured as 16.70 ± 4.17 μA (RSD%,

24.98%, n=4) and 20.36 ± 10.68 µA (RSD%, 52.46%, n=4) after hybridization of Probe-1 with miRNA-155 target during 5 min and 15 min, respectively. According to the signal measured in the absence of target, the highest decrease (28.9%) was recorded in the case of 5 min hybridization time. Thus, it was chosen as optimum hybridization time for further studies.

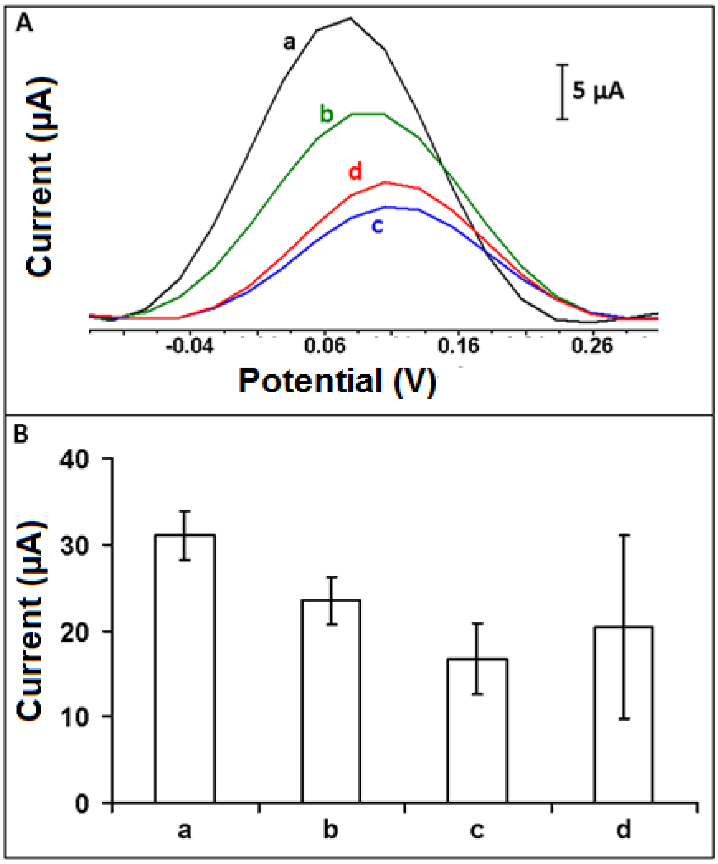

Figure S6. (A) DPVs, **(B)** histograms representing the $[Fe(CN)_6]^{3-/4-}$ oxidation signal obtained by (a) AuNPs/RGO-modified paper electrode, (b) 0.5 µg/mL Probe-1 immobilized AuNPs/RGO-modified paper electrode, after the hybridization of Probe-1 with miRNA-155 target during (c) 5 min, (d) 15 min (n=4).

Table S3. The oxidation signal of $[Fe(CN)_6]^{3-/4-}$ measured before/after hybridization of Probe-1 and miRNA-155 target in its different concentrations (n=3) and HE% values.

		**I (µA)**	
**Probe-1 immobilized AuNPs/RGO-modified paper electrode**		26.79 ± 2.42	**HE%**
**[miRNA-155] (µg/mL)**	0.25	24.69 ± 0.34	7.8%
	0.5	22.34 ± 3.06	16.6%
	0.75	18.01 ± 0.20	32.7%
	1	16.83 ± 1.91	37.1%
	1.5	16.94 ± 0.76	36.7%
	2	17.25 ± 0.95	35.6%

Table S4. The oxidation signal of $[Fe(CN)_6]^{3-/4-}$ and decrease % at the signal after hybridization of Probe-2 and miRNA-21 target in its different concentrations (n=3).

		**I (μA)**	
**Probe-2 immobilized AuNPs/RGO-modified paper electrode**		29.70 ± 0.20	**HE%**
**[miRNA-21] (μg/mL)**	0.25	27.36 ± 3.59	7.8%
	0.5	23.40 ± 2.96	21.2%
	0.75	20.73 ± 2.34	30.2%
	1	16.85 ± 1.09	43.2%
	1.5	17.22 ± 0.65	42.0%
	2	17.16 ± 0.08	42.2%

Selectivity of the assay on voltammetric detection of miRNA-155 by AuNPs/RGO-modified paper electrode

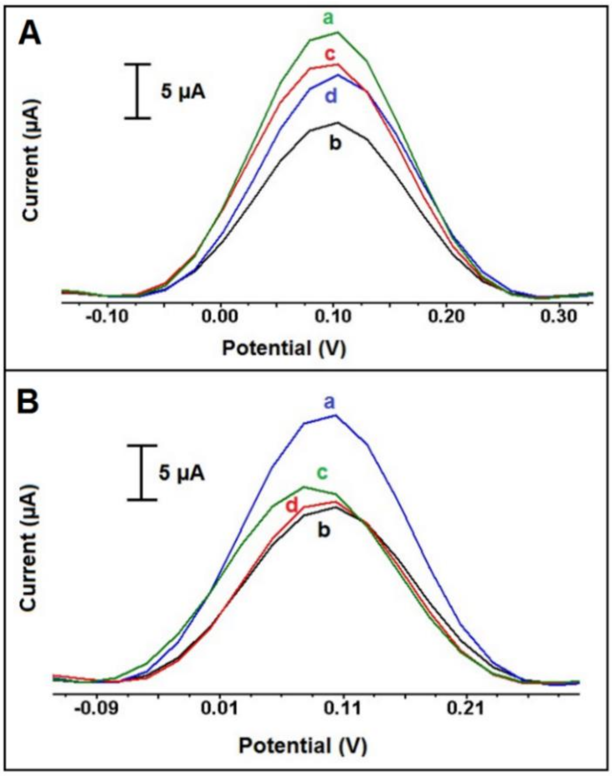

Figure S7. (A) Voltammograms representing the $[Fe(CN)_6]^{3-/4-}$ oxidation signal obtained by (a) Probe-1 immobilized AuNPs/RGO-modified paper electrode in the absence of miRNA-155 target, after hybridization of Probe-1 with (b) miRNA-155 target, (c) NC, and (d) MM, individually. **(B)** Voltammograms representing the $[Fe(CN)_6]^{3-/4-}$ oxidation signal obtained by (a) Probe-1 immobilized AuNPs/RGO-modified paper electrode in the absence of miRNA-155 target, after hybridization of

Probe-1 (b) with only miRNA-155 target, (c) in target:NC (1:1) mixture, and (d) in target:MM (1:1) mixture.

Table S5. The average $[Fe(CN)_6]^{3-/4-}$ oxidation signals measured before and after hybridization of Probe-1 with miRNA-155 target, NC, MM, the mixture sample containing target:NC (1:1) or the mixture sample containing target:MM (1:1). HE% calculated according to the oxidation signals obtained after hybridization.

	**I (µA)**	
**Probe-1 immobilized AuNPs/RGO-modified paper electrode**	29.47 ± 0.44 µA	**HE%**
**miRNA-155 target**	17.32 ± 3.22 µA	41.2%
**NC**	20.05 ± 2.35 µA	31.9%
**MM**	20.04 ± 2.71 µA	31.9%
**miRNA-155 target:NC (1:1) mixture**	18.40 ± 1.62 µA	37.5%
**miRNA-155 target:MM (1:1) mixture**	21.67 ± 5.12 µA	26.4%

Selectivity of the assay on voltammetric detection of miRNA-21 by AuNPs/RGO-modified paper electrode

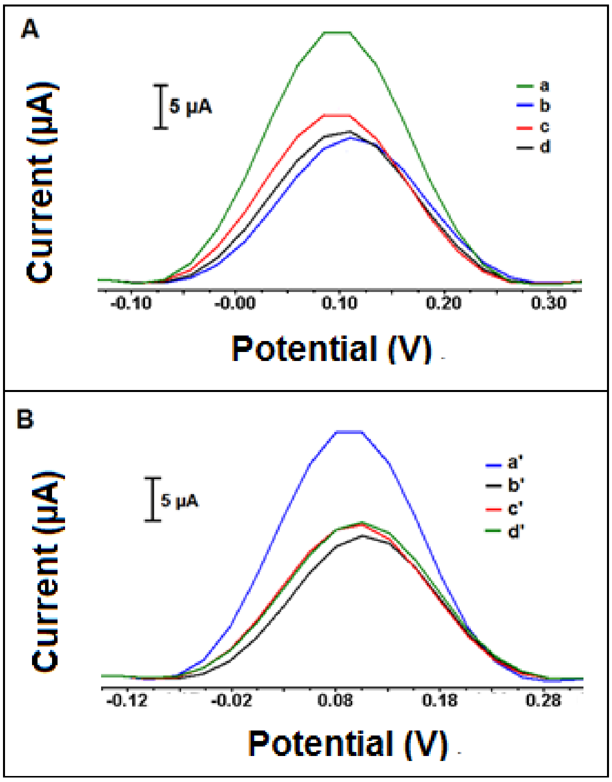

Figure S8. (A) Voltammograms representing the $[Fe(CN)_6]^{3-/4-}$ oxidation signal obtained by (a) Probe-2 immobilized AuNPs/RGO-modified paper electrode in the absence of miRNA-21 target, after hybridization of Probe-2 with (b) miRNA-21 target, (c) NC, and (d) MM, individually. **(B)** Voltammograms representing the $[Fe(CN)_6]^{3-/4-}$ oxidation signal obtained by (a') Probe-2 immobilized AuNPs/RGO-modified paper electrode in the absence of miRNA-21 target, after hybridization of Probe-2 (b') with only miRNA-21 target, (c') in target:NC (1:1) mixture, and (d') in target:MM (1:1) mixture

Table S6. The average $[Fe(CN)_6]^{3-/4-}$ oxidation signals measured before and after hybridization of Probe-2 with miRNA-21 target, NC, MM, the mixture sample containing target:NC (1:1) or the mixture sample containing target:MM (1:1). HE % calculated according to the oxidation signals obtained after hybridization.

	**I (µA)**	
**Probe-2 immobilized AuNPs/RGO-modified paper electrode**	25.83±5.08 µA	**HE%**
**miRNA-21 target**	17.00±3.17 µA	34.1%
**NC**	20.64±5.75 µA	20.1%
**MM**	18.65±4.12 µA	27.8%
**miRNA-21 target:NC (1:1) mixture**	17.65±4.09 µA	31.7%
**miRNA-21 target:MM (1:1) mixture**	18.44±4.09 µA	28.6%

Results obtained by AuNPs/MoS$_2$-modified paper electrode

The electrochemical characterization of AuNPs/MoS$_2$-modified paper electrode

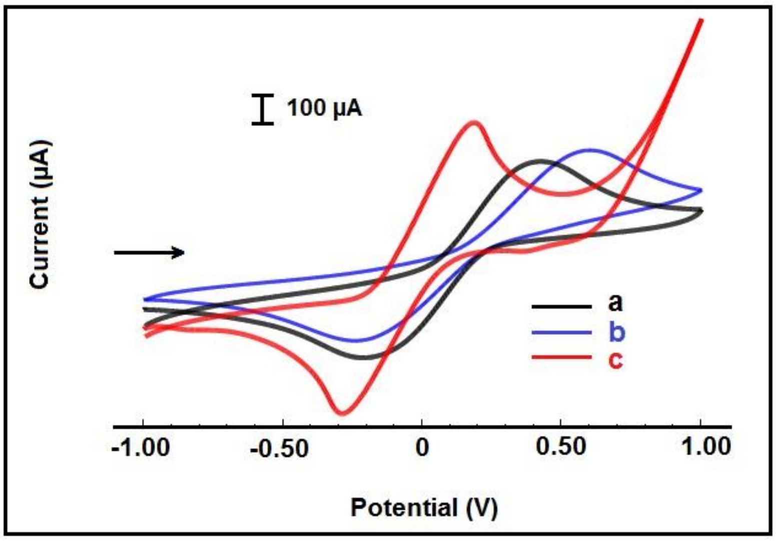

Figure S9. CVs recorded in optimum conditions by (a) unmodified paper electrode, (b) MoS_2-modified paper electrode, (c) AuNPs electrodeposited MoS_2-modified paper electrodes in the presence of 50.0 mM potassium ferricyanide in 100.0 mM KCl.

Table S7. The anodic current Ia (µA) and the cathodic current Ic (µA), the relative charge, Qa and Qc of $[Fe(CN)_6]^{3-/4-}$ measured by unmodified, MoS_2 modified and AuNPs/MoS_2-modified paper electrode.

	**Ia (µA)**	**Ic (µA)**	**Qa (mC)**	**Qc (mC)**	**A (cm^2)**
**Unmodified paper electrode**	236.15	241.36	2.23	3.10	0.020
**MoS_2-modified paper electrode**	245.23	280.60	2.21	3.91	0.021
**AuNPs/MoS_2-modified paper electrode**	415.77	448.02	4.40	4.91	0.035

The effect of probe concentration at hybridization process:

The effect of DNA probe concentration upon the hybridization was investigated (Fig. S10). The nucleic acid hybridization was performed between 1.0 µg/mL miRNA-155 target and its Probe-1 at the different concentration level from 0.5, and 1.0 µg/mL onto the surface of AuNPs/MoS_2-modified paper electrode. After immobilization of 0.5 µg/mL Probe-1, the average oxidation signal of $[Fe(CN)_6]^{3-/4-}$ was measured as 28.39 ± 0.94 µA (RSD%, 3.32%, n=5), whereas the oxidation signal was measured as 32.06 ± 0.76 µA (RSD%, 2.36%, n=3) after immobilization of 1.0 µg/mL Probe-1. After hybridization of 0.5 µg/mL or 1.0 µg/mL Probe-1 with miRNA-155 target the oxidation signals were measured as 22.61 ± 0.62 µA (RSD%, 2.74%, n=3) and 24.59 ± 2.79 µA (RSD%, 11.35%, n=3), respectively. According to the more reproducible results, 0.5 µg/mL Probe-1 concentration was determined as optimum for further studies.

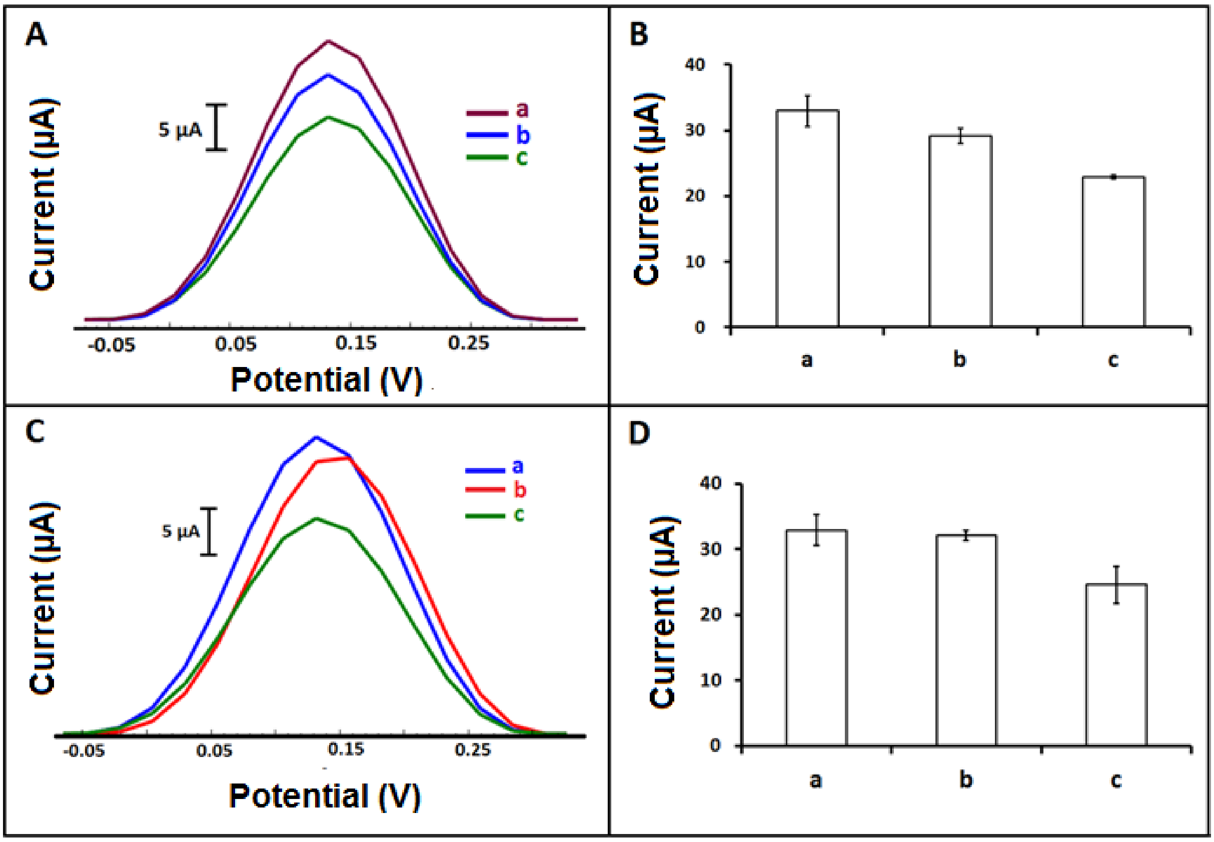

Figure S10. (A) DPVs **(B)** histograms representing (a) AuNPs/MoS_2-modified paper electrode, (b) 0.5 µg/mL DNA probe immobilized AuNPs/MoS_2-modified paper electrode, (c) after hybridization of 0.5 µg/mL DNA probe with 1.0 µg/mL miRNA-155 target (n=2). **(C)** DPVs **(D)** histograms representing (a) AuNPs/MoS_2-modified paper electrode, (b) 1.0 µg/mL DNA probe immobilized AuNPs/MoS_2-modified paper electrode, (c) after hybridization of 1.0 µg/mL DNA probe with 1.0 µg/mL miRNA-155 target (n=2).

The effect of hybridization time upon the hybridization process

The hybridization of 0.5 µg/mL Probe-1 and 1.0 µg/mL miRNA-155 target was done during 5 and 15 min hybridization time (Fig. S11). The average oxidation signal of $[Fe(CN)_6]^{3-/4-}$ was measured as 22.95 ± 0.30 µA (RSD%, 1.33%, n=2) and 30.55 ± 7.40 µA (RSD%, 24.24%, n=3) after hybridization of Probe-1 with miRNA-155 target during 5 min and 15 min, respectively. According to the signal obtained in the absence of target, the highest decrease as 32% was obtained in case of 5 min hybridization time. Thus, 5 min hybridization time was chosen as optimum for further studies.

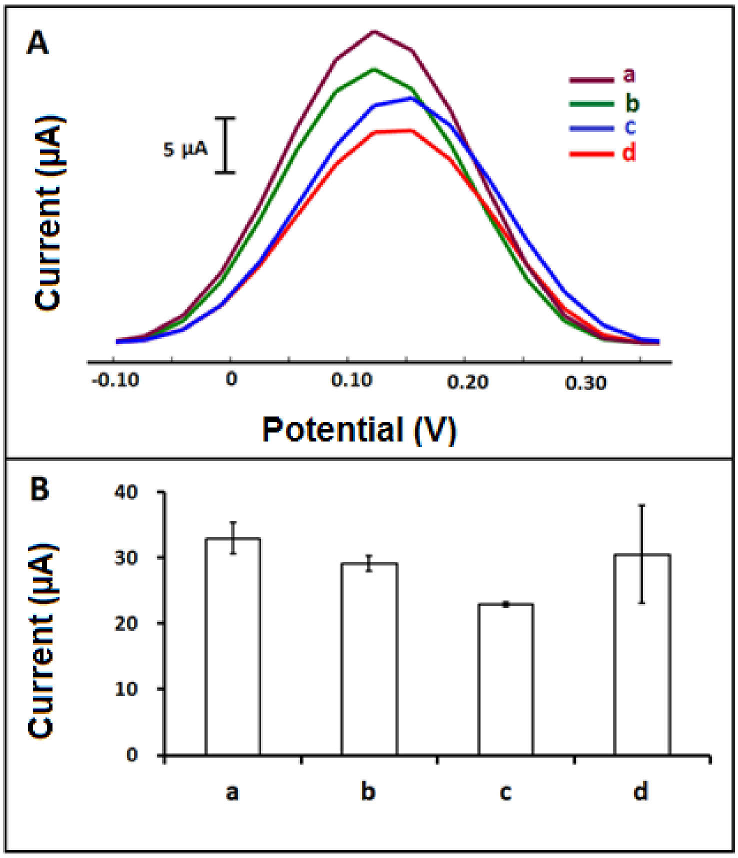

Figure S11. (A) DPVs, **(B)** histograms representing the $[Fe(CN)_6]^{3-/4-}$ oxidation signal obtained by (a) AuNPs/MoS_2-modified paper electrode, (b) 0.5 µg/mL Probe-1 immobilized AuNPs/MoS_2-modified paper electrode, after the hybridization of Probe-1 with miRNA-155 target during (c) 5 min, (d) 15 min (n=3).

Voltammetric detection of miRNA-21 by AuNPs and MoS_2-modified paper electrode

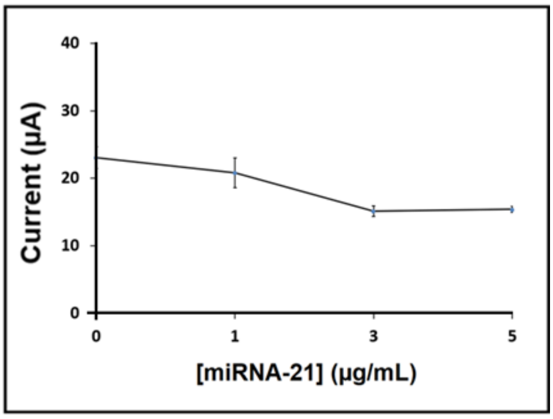

Figure S12. The line graph based on the average $[Fe(CN)_6]^{3-/4-}$ oxidation signal measured after hybridization between Probe-2 and miRNA-21 target with its various concentrations from 0 to 5.0 µg/mL (n=3).

Tablo S8. The oxidation signal of $[Fe(CN)_6]^{3-/4-}$ and HE% values that calculated after hybridization of Probe-2 and miRNA-21 target in its different concentrations (n=3).

		**I (μA)**	
**Probe-2 immobilized $AuNPs/MoS_2$-modified paper electrode**		23.06 ± 1.59	**HE%**
**[miRNA-21] (μg/mL)**	0.5	22.48 ± 1.62	2.5%
	1	20.80 ± 2.21	9.8%
	1.5	19.14 ± 1.92	16.9%
	2	17.35 ± 0.65	24.7%
	2.5	16.69 ± 0.92	27.6%
	3	15.13 ± 0.76	34.3%
	5	15.38 ± 0.45	33.3%

Voltammetric detection of miRNA-155 by AuNPs and MoS_2-modified paper electrode

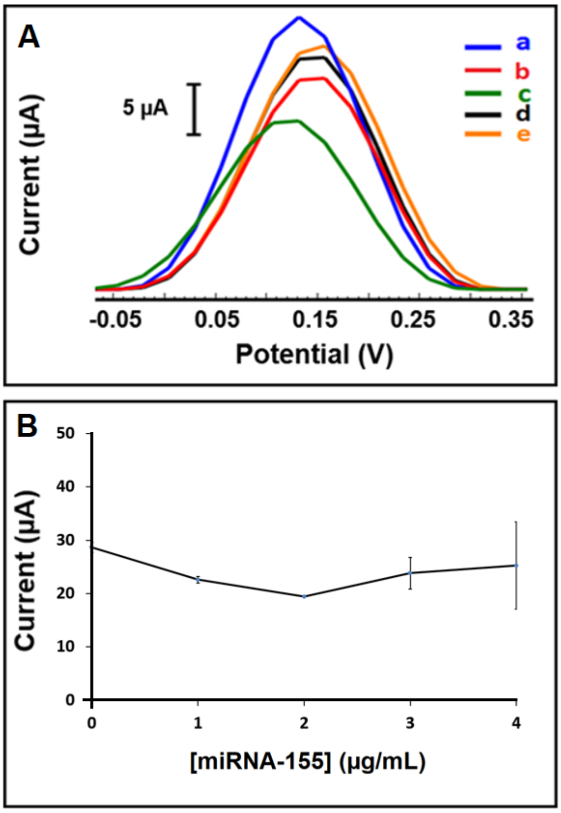

Figure S13. (A) Voltammograms representing the $[Fe(CN)_6]^{3-/4-}$ oxidation signals obtained by (a) Probe-1 immobilized $AuNPs/MoS_2$-paper electrode, after hybridization of Probe-1 with miRNA-155 target at the concentrations of (b) 1.0 μg/mL, (c) 2.0 μg/mL, (d) 3.0 μg/mL, (e) 4.0 μg/mL. **(B)** The line graph based on the average $[Fe(CN)_6]^{3-/4-}$ oxidation signal after hybridization between Probe-1 and miRNA-155 target with its various concentrations from 0 to 4.0 μg/mL (n=3).

Table S9. The oxidation signal of $[Fe(CN)_6]^{3-/4-}$ and HE% values that calculated after hybridization of Probe-1 and miRNA-155 target in its different concentrations (n=3).

		I (μA)	
**Probe-1 immobilized AuNPs/MoS_2-modified paper electrode**		28.65 ± 0.94	**HE%**
**[miRNA-155] (μg/mL)**	1	22.61 ± 0.62	21.0%
	2	19.38 ± 0.04	32.3%
	3	23.80 ± 2.98	16.9%
	4	25.24 ± 8.17	11.9%

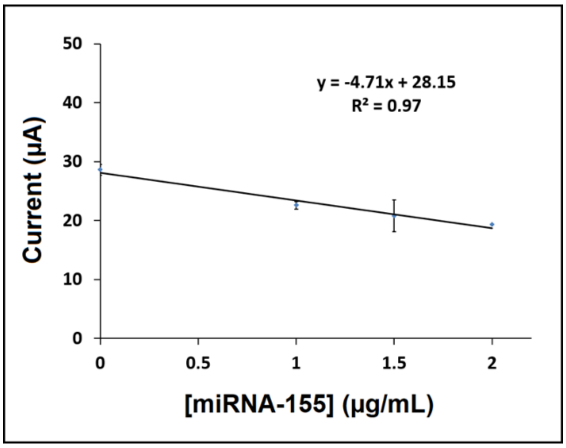

Figure S14. The calibration plot obtained after hybridization between Probe-1 and miRNA-155 target with its various concentrations from 0 to 2.0 μg/mL (n=3).

Selectivity of the assay on voltammetric detection of miRNA-155 by AuNPs and MoS_2-modified paper electrode

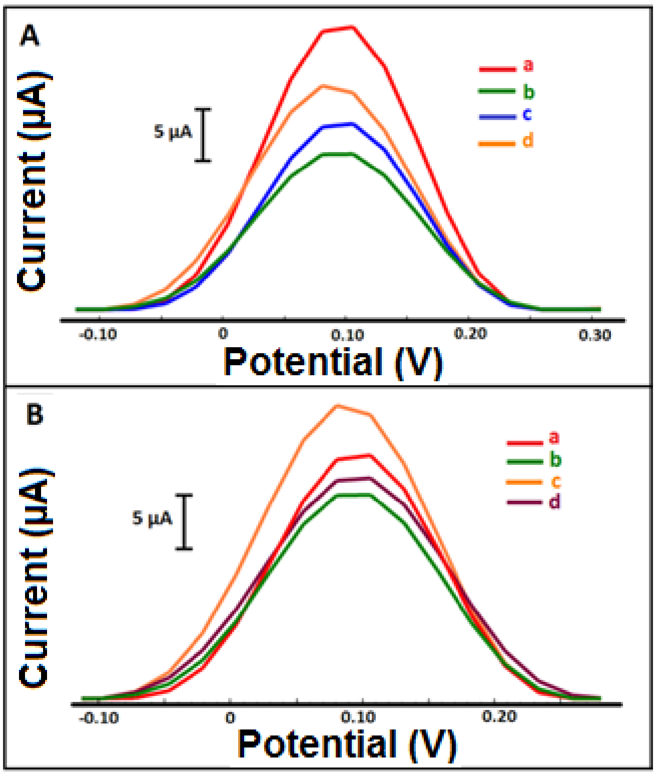

Figure S15. (A) Voltammograms representing the $[Fe(CN)_6]^{3-/4-}$ oxidation signal obtained by (a) Probe-1 immobilized AuNPs/MoS_2-modified paper electrode in the absence of miRNA-155 target, after hybridization of Probe-1 with (b) miRNA-155 target, (c) NC, and (d) MM, individually. **(B)** Voltammograms representing the $[Fe(CN)_6]^{3-/4-}$ oxidation signal obtained by (a) Probe-1 immobilized AuNPs/MoS_2-modified paper electrode in the absence of miRNA-155 target, after hybridization of Probe-1 (b) with only miRNA-155 target, (c) in target:NC (1:1) mixture, and (d) in target:MM (1:1) mixture.

Table S10. The average $[Fe(CN)_6]^{3-/4-}$ oxidation signals (n=2) measured before and after hybridization of Probe-1 with miRNA-155 target, NC, MM, the mixture sample containing target:NC (1:1) or the mixture sample containing target:MM (1:1). HE% calculated according to the oxidation signals obtained after hybridization.

	**I (µA)**	
**Probe-1 immobilized AuNPs/MoS_2-modified paper electrode**	28.39 ± 0.94	**HE%**
**miRNA-155 target**	19.74 ± 1.75	30.5 %
**NC**	30.77 ± 8.37	8 %
**MM**	22.52 ± 2.80	20.6 %
**miRNA-155 target:NC (1:1) mixture**	20.59 ± 2.59	27.5 %
**miRNA-155 target:MM (1:1) mixture**	21.23 ± 3.00	25.2 %

Selectivity of the assay on voltammetric detection of miRNA-21 by AuNPs and MoS_2-modified paper electrode

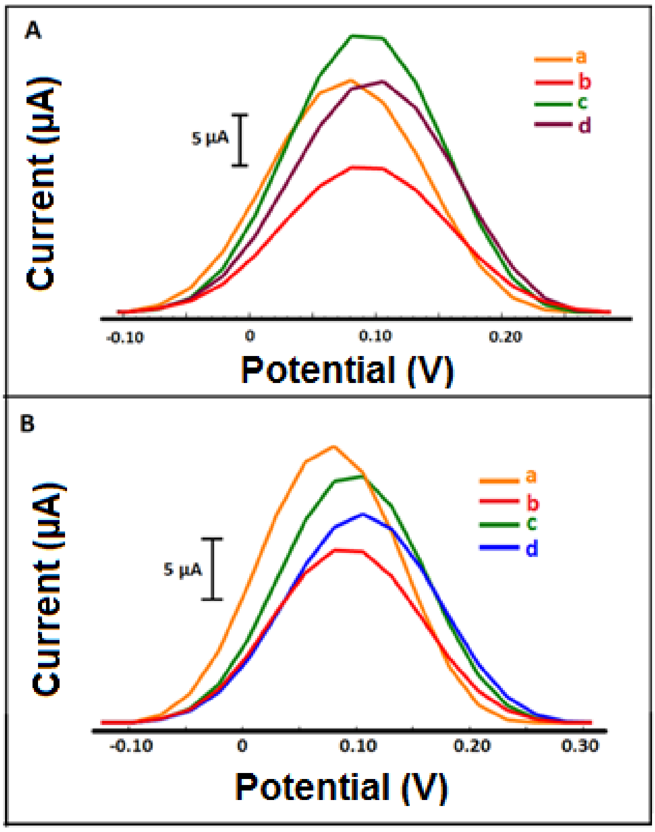

Figure S16. (A) Voltammograms representing the $[Fe(CN)_6]^{3-/4-}$ oxidation signal obtained by (a) Probe-2 immobilized AuNPs/MoS_2-modified paper electrode in the absence of miRNA-21 target, after hybridization of Probe-2 with, (b) miRNA-21 target, (c) NC, and (d) MM, individually. **(B)** Voltammograms representing the $[Fe(CN)_6]^{3-/4-}$ oxidation signal obtained by (a) Probe-2 immobilized AuNPs/MoS_2-modified paper electrode in the absence of miRNA-21 target, after hybridization of Probe-2 (b) with only miRNA-21 target, (c) target:NC (1:1) mixture, and (d) in target:MM (1:1) mixture.

Table S11. The average $[Fe(CN)_6]^{3-/4-}$ oxidation signals (n=2) measured before and after hybridization of Probe-2 with miRNA-21 target, NC, MM, the mixture sample containing target:NC (1:1) or the mixture sample containing target:MM (1:1). HE% calculated according to the oxidation signals obtained after hybridization.

	**I (μA)**	
**Probe-2 immobilized AuNPs/MoS_2-modified paper electrode**	23.06 ± 1.59	**HE%**
**miRNA-21 target**	16.20 ± 3.12	29 %
**NC**	27.13 ± 3.01	17 %
**MM**	24.54 ± 3.92	6 %
**miRNA-21 target:NC (1:1) mixture**	21.66 ± 4.79	6 %
**miRNA-21 target:MM (1:1) mixture**	21.88 ± 5.24	5%
